# Association mapping in sunflower for sclerotinia head rot resistance

**DOI:** 10.1186/1471-2229-12-93

**Published:** 2012-06-18

**Authors:** Corina M Fusari, Julio A Di Rienzo, Carolina Troglia, Verónica Nishinakamasu, María Valeria Moreno, Carla Maringolo, Facundo Quiroz, Daniel Álvarez, Alberto Escande, Esteban Hopp, Ruth Heinz, Verónica V Lia, Norma B Paniego

**Affiliations:** 1 Instituto de Biotecnología, Centro Investigación en Ciencias Veterinarias y Agronómicas (CICVyA), Instituto Nacional de Tecnología Agropecuaria (INTA), 1686, Hurlingham, Buenos Aires, Argentina; 2 Cátedra de Estadística y Biometría, Facultad de Ciencias Agropecuarias, Universidad Nacional de Córdoba, 5000, Córdoba, Argentina; 3 Estación Experimental Agropecuaria Balcarce, INTA, 7620, Balcarce, Buenos Aires, Argentina; 4 Estación Experimental Agropecuaria Manfredi, INTA, 5988, Manfredi, Córdoba, Argentina; 5 Facultad de Ciencias Exactas y Naturales, Universidad de Buenos Aires, Buenos Aires, Argentina

## Abstract

**Background:**

Sclerotinia Head Rot (SHR) is one of the most damaging diseases of sunflower in Europe, Argentina, and USA, causing average yield reductions of 10 to 20 %, but leading to total production loss under favorable environmental conditions for the pathogen. Association Mapping (AM) is a promising choice for Quantitative Trait Locus (QTL) mapping, as it detects relationships between phenotypic variation and gene polymorphisms in existing germplasm without development of mapping populations. This article reports the identification of QTL for resistance to SHR based on candidate gene AM.

**Results:**

A collection of 94 sunflower inbred lines were tested for SHR under field conditions using assisted inoculation with the fungal pathogen *Sclerotinia sclerotiorum*. Given that no biological mechanisms or biochemical pathways have been clearly identified for SHR, 43 candidate genes were selected based on previous transcript profiling studies in sunflower and *Brassica napus* infected with *S. sclerotiorum*. Associations among SHR incidence and haplotype polymorphisms in 16 candidate genes were tested using Mixed Linear Models (MLM) that account for population structure and kinship relationships. This approach allowed detection of a significant association between the candidate gene *HaRIC_B* and SHR incidence (*P* < 0.01), accounting for a SHR incidence reduction of about 20 %.

**Conclusions:**

These results suggest that AM will be useful in dissecting other complex traits in sunflower, thus providing a valuable tool to assist in crop breeding.

## Background

Sunflower (*Helianthus annuus* L.) is one of the most important sources of vegetable oil grown worldwide [1]. Fungal infections represent one of the main constraints for crop yield and productivity, having a detrimental impact on quality components. *Sclerotinia sclerotiorum* (Lib) de Bary is a worldwide distributed necrotrophic pathogen, attacking more than 400 plant species including sunflower, soybean and rapeseed [2,3]. The fungus can attack several plant organs causing diverse symptoms in leaves, stalks and flowers, with Sclerotinia Head Rot (SHR) being the most damaging for sunflower crop production. SHR is considered a major disease in Europe, Argentina, and USA, causing average yield reductions of 10 to 20 %, and can even result in loss of the entire harvest [4,5]. Disease control on *S. sclerotiorum* is difficult, since the fungus persists in soils for long periods and at high inoculum levels [6-8].

Resistance to *S. sclerotiorum* has been described as quantitatively inherited with predominantly additive gene action, and medium heritability [3]. Classical linkage mapping based on biparental populations was used to dissect Quantitative Trait Loci (QTL) for SHR resistance. These analyses have rendered QTL with small effects, explaining only a minor proportion of the phenotypic variance [9-15]. In addition, a number of studies have been done in other *S. sclerotiorum* host species to understand the defense mechanisms triggered in resistant genotypes [16-25]. One of the most comprehensive studies has been conducted in *Brassica napus* by performing microarray analysis in resistant and susceptible genotypes infected with *S. sclerotiorum*[25]. A total of 686 and 1,586 genes were found to be differentially expressed after infection in the resistant and susceptible genotypes, respectively. The number of differentially expressed genes increased over infection time, most of them being up-regulated. The putative functions of these genes included pathogenesis related proteins, proteins involved in oxidative burst, protein kinase and molecule transporters, among others [25]. However, extrapolating this information to sunflower, and using it to evaluate new sources of resistance requires the identification of orthologous genes between *B. napus* and *H. annuus*.

Association Mapping (AM) was suggested as a promising alternative to classical linkage mapping to elucidate the genetic basis of complex traits [26]. The AM approach is based on the extent of Linkage Disequilibrium (LD) observed in a set of accessions that are not closely related. In contrast to classical biparental population mapping, AM is a method that detects relationships between phenotypic variation and gene polymorphisms in existing germplasm, without development of mapping populations. This method incorporates the effects of recombination occurring in many past generations into a single analysis and thus, it is complementary to the classical biparental approach [27]. The main drawback of AM is the possibility of false-positive results due to unrecognized population structure. In order to avoid population stratification effects, information on the relatedness among genotypes is commonly included trough the recognition of the population structure and/or as a kinship matrix between genotypes [26].

AM has been successfully applied in mapping genes involved in several traits in different plant species (e.g. flowering time and aluminum tolerance in maize, resistance to late blight in potato, kernel size and milling quality in wheat, resistance to dieback in lettuce) [28-32]. Even though two possible strategies have been proposed, Genome Wide Association (GWA) and candidate gene approaches, the latter has been the most widely used in plants, mostly due to the lack of complete genome data for many plant species.

Despite the fact that both nucleotide diversity and decay of LD have been assessed in sunflower germplasm from different origins [33-35], no AM studies have been published to date either for SHR or any other trait.

This paper reports the identification of resistance QTL for SHR based on candidate gene AM. Given that no biological mechanisms or biochemical pathways have been positively identified for SHR, selection of candidate genes was based on previous transcript profiling studies in sunflower [36-38] and *B. napus*[25]. This approach resulted successful in detecting a significant association between one of the candidate genes evaluated and SHR incidence. These results suggest that AM is a useful strategy for dissecting complex traits in sunflower, thus providing a valuable tool to assist in crop breeding.

## Results

### Phenotypic data

**Figure 1:**
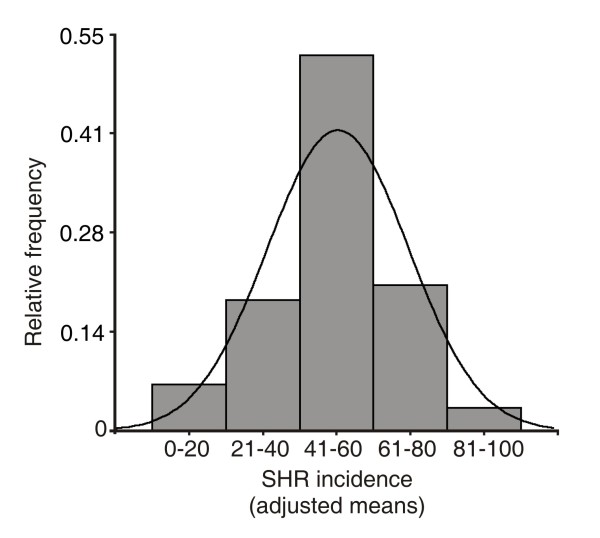
**Sclerotinia Head Rot incidence.** Phenotypic behavior of the AMP measured as the adjusted means of SHR incidence.

Ninety-four inbred lines belonging to the “Sunflower Breeding Program” of INTA were evaluated for SHR incidence during 2008/2009 and 2009/2010 in replicated trials. Because the experiments were conducted as randomized complete blocks, and repeated in two consecutive trials, the model used to obtain the adjusted line means, included the trial, and the blocks within trials, as random effects. All inoculation days were suitable to produce disease. The line effect was significant (*P* < 0.0001). The adjusted SHR incidence means varied form 0 % to 100 %, with an average of 50.4 %. In fact, 52 % of the Association Mapping Population (AMP) showed an intermediate behavior against the disease, i.e. between 40 % and 60 % (Figure 1 and Additional file 1).

### Candidate gene selection

A total of 43 genes were used as starting point for candidate gene selection. Putative orthologous sequences, either from sunflower or from other Asteraceae species were identified for 18 out of 19 *A. thaliana* loci selected from the work of Zhao et al. [25] using the phylogenetic approach detailed in Methods. After PCR amplification using sunflower genomic DNA as template, products with the expected sequence were obtained for 10/19 loci. For six of them, paralogous sequences were also identified in *Helianthus*. Thus, the initial 10 loci of *A. thaliana* correlated with 17 genes in sunflower (*HaMTHaPRP1HaPRP2HaCYP450AHaCYP450BHaCYP450CHaROPGEF1HaROPGEF2HaROPGEF3HaPP2CHaRIC_AHaRIC_BHaHP1HaHP2HaFbox1HaFbox2* and *HaZFHD*) (Table 1).

**Table 1:** Candidate genes for SHR: sources of selection and features for genotyping the association mapping population

**Source of selection**	**Target locus**	**Sunflower gene ID**^**a**^	**Sequence length**^**b**^	**SNPs in CS**^**c**^	**Indels in CS**^**d**^	**Haplotypes in CS**^**e**^	**Genotyping method**^**f**^	**Primers for AMP genotyping**
*B. napus* expression	At1g34580	* HaMT *	580 (191)	8 (8)	3 (11)	3 (2)	FCE	F:5′GAAAGGACATGCTACTTTATGG3′
								R:5′CTTTACTTGAATTAAAGTTACT3′
profile	At5g51550	*HaPRP1*	431	0	-	1		
		* HaPRP2 *	437 (166)	11 (11)	-	3 (2)	dHPLC	F: 5′GGACGGGAACGTAAAATAATG3′
								R: 5′CCGTCTGTCCGTACAATCG3′
	At3g48310	*HaCYP450A*	786	1 (0)	-	2		
		*HaCYP450B*	310	2 (0)	-	2		
		* HaCYP450C *	400 (260)	2 (2)	1 (1)	2 (6)	dHPLC	F: 5′AAGTGACTTTAGCAACGTCC3′
								R: 5′GAGTTGGTATGGGTGGATGAA3′
	At5g05940	*HaROPGEF1*	447	12 (11)	3 (50)	4		
		* HaROPGEF2 *	403 (197)	2 (2)	2 (11)	2 (2)	FCE	F: 5′TGCGTAGTGGTTCTAAAATTGG3′
								R: 5′CGTCAATCATTACCCCAACC3′
		*HaROPGEF3*	801	0	-	1		
	At1g48040	* HaPP2C *	514 (398)	4 (4)	-	2 (4)	Direct sequencing	F: 5′ACTGGGACTACGGCATTGAC3′
								R: 5′TGCTGAATTTCTGGCTCTGA3′
	At1g04450	* HaRIC_A *	940 (261)	8 (8)	4 (6)	3 (2)	FCE	F: 5′GCACGAATAGTGACATTGAAAC3′
								R:5′ACATAAAACAGTTTTCGGTCC3′
		* HaRIC_B *	1424 (537)	12 (0)	2 (15)	3 (3)	FCE	F: 5′GGCTTGCGTTACATCTCTGA3′
								R: 5′CCCAACTAGGAGCATTGGAA 3′
	At1g03687	*HaHP1*	198	0	1(1)	1		
	At4g09180	* HaHP2 *	309 (256)	1 (1)	-	2 (5)	Direct sequencing	F: 5′CTGCTATCCAGGCTCATTCA3′
								R: 5′AGAATGGCAGGGCGACCAAG3′
	At1g13200	*HaFbox1*	265	11 (11)	-	3		
		*HaFbox2*	262	8 (5)	1 (1)	5		
	At1g14687	* HaZFHD *	166 (166)	9 (7)	-	4 (9)	Direct sequencing	F:5′TCATGCCCTCACTAACATGC 3′
								R: 5′TTTGTCCGGAATCTTTTTCG 3′
Sunflower EST library	TC49193	*HaDRP*	587	14 (12)	1 (1)	2		
	BQ973243	*HaCP*	542	14 (10)	-	3		
	-	* HaGDPDI *	620 (707)	18 (14)	2 (2)	3 (10)	Direct sequencing	F: 5′CAGAAACTGATCAACCCGAAA 3′
								R: 5′TGCATGCATCTTGGAAAATAG 3′
	-	* HaCWP *	351 (177)	4 (4)	3 (10)	2 (2)	FCE	F: 5′CAGGAATCACGGTCCCTAGT3′
								R: 5′TGAAACATGAGGGATGAGCA3′
	TC42391	* HaPI *	658 (235)	14 (9)	3 (4)	5 (2)	FCE	F: 5′TCCAACAGTGTGTGACCTTTG3′
								R: 5′CATTAGTTACGTTACAAAGCTAT3′
	TC57179	* HaPAL *	343 (343)	2 (2)	-	2 (2)	dHPLC	F:5′TGTGGTCTTCAAATTCATTAATAAC3′
								R: 5′GGCCATTCCTAACAGGATCA3′
Defense responses	CF088675	* HaWRKY5 *	1395 (227)	39 (33)	14 (56)	8 (6)	FCE	F: 5′CCGATCAAAGGCTCAATCTA3′
								R: 5′CACATCCGCTAGTTCACACC3′
	BU016906	* HaWRKY7 *	1163 (174)	1 (1)	2 (22)	3 (2)	FCE	F: 5′CATTGTTGGTCAACCCTGTG3′
								R: 5′AGGGAAGCATAACCATGACG3′
	EU112647	*HaGLP1*	876	0	3 (3)	3		
	AJ540203	*HaGLP2*	794	6 (6)	-	2		
	TC17527	*HaGLP3*	621	13 (9)	-	6		
	TC18217	* HaGLP4 *	761 (209)	9 (2)	3 (11)	4 (4)	FCE	F: 5′TGGCTGCAACAACTTTCCTT3′
								R: 5′TTCAATCCAGAAACAAACTTCTAA3′
	TC17648	*HaGLP5*	1710	2 (1)	2 (3)	3		

^a^ Acronyms used throughout this article, candidate genes genotyped in the AMP are underlined.

^b^ Length of PCR products sequenced in the CS. Length of PCR products genotyped in the AMP are given in parentheses.

^c^ Parsimony informative sites are given in parentheses.

^d^ Base pairs of indels are given in parentheses.

^e^ Number of haplotypes detected in the AMP are given in parentheses.

^f^ FCE: fluorescent capillary electrophoresis, dHPLC: denaturing high liquid performance chromatography.

^g^ Primers 5’-end 6-FAM labeled.

Specific PCR products were obtained for 11/17 genes selected from the RHA801 EST library (*HaDRP*, *HaCP*, *HaGDPDI*, *HaCWP*, *HaPI*, *HaPAL*, *HaRNAHe*, *HaHP3*, *HaKIV*, *HaPEP*, *HaTF1*). Conversely, PCR amplification of *Ha26SPS*, *HaTF2* and *HaLTP1* yielded unspecific products, whereas little or no amplification was observed for *HaTHI*, *HaTE*, *HaPK*. Introns of variable length (500–1200 pb) were found for *HaRNAHe*, *HaHP3*, *HaKIV*, *HaPEP* and *HaTF1*.

Five germin-like proteins (*HaGLP1* to *HaGLP5*) and two transcription factors from the WRKY family (*HaWRKY5* and *HaWKRY7*) were amplified successfully. Summarizing, a total of 30 candidate genes were suitable for amplification in the 10 sunflower inbred lines selected as Core Set (CS) for initial polymorphism development. The candidates encompassed 17 genes derived from the expression analysis of Zhao et al. [25], six genes from the RHA801 EST library [38] and seven genes chosen based on their putative role in defense mechanisms in sunflower [36,37] (Table 1).

### SNP identification and genotyping of candidate genes

Polymorphisms were found in 28/30 candidate genes, and 21 were further selected to be genotyped in the AMP. The seven genes that were not included in subsequent analyses were discarded due to (1) minor allele frequencies lower than 0.2 in the CS (*HaCYP450A*, *HaCYP450B* and *HaHP1*), (2) failure of DNA sequencing in some members of the CS (*HaROPGEF1*, *HaFbox1* and *HaFbox2*) and (3) the instability of polymorphic sites (e.g. indels made of poly-T tracts in *HaGLP1*, Table 1). Genes *HaDRP*, *HaCP*, *HaGLP2*, *HaGLP3* and *HaGLP5*, selected to be genotyped by denaturing High Performance Liquid Chromatography (dHPLC) in the AMP, did not achieve the expected resolution and were also excluded from further analyses. Hence, 16/28 polymorphic candidate genes were genotyped in the AMP. Three candidate genes were genotyped by dHPLC (*HaPRP2*, *HaCYP450C*, *HaPAL*), nine by Fluorescent Capillary Electrophoresis (FCE) (*HaMT*, *HaROPGEF2*, *HaRIC_A*, *HaRIC_B*, *HaCWP*, *HaPI*, *HaWRKY5*, *HaWRKY*, *HaGLP4*) and four were typified by direct sequencing (*HaPP2C*, *HaHP2*, *HaZFHD*, *HaGDPDI*) (Table 1). Haplotypes not present in the CS were found in the AMP for *HaCYP450C*, *HaPP2C*, *HaRIC_B*, *HaHP2*, *HaZFHD*, *HaGDPDI* and *HaWRKY5*. Regarding the 16 candidate genes genotyped in the AMP, the number of haplotypes ranged from two to nine, with an average of 3.6 haplotypes per gene. The average haplotype frequency was 27.6 %. The lowest haplotype frequency (1.1 %) was found for *HaCYP450C* and *HaWRKY5*, whereas the highest haplotype frequency (93.4 %) was found for *HaPI* gene.

### Population structure

The AMP was characterized using eight SSRs, resulting in the detection of 47 alleles, ranging from four alleles at locus HA4103 to nine at locus HA991 (average of 6.6 alleles per locus).

Population structure was evaluated through Principal Coordinate Analysis (PCO) and the Bayesian method implemented in STRUCTURE software [39]. The first two principal coordinates of the PCO explained 12.6 % and 9.8 % of the molecular variance, with no clear clustering of individuals being detected among the 94 inbred lines analyzed (Additional file 2). According to the STRUCTURE analysis, the log-likelihood values of the data conditional on k reached a plateau at k = 9 (Additional file 2: Figure S1B). Likewise, the *ad hoc* procedure proposed by Evanno et al. [40] showed peaks of Δk at k = 9, k = 5 and k = 2. Thus, k = 9 was selected as the most likely number of ideal populations, since its signal was detected by both the log-likelihood and the Evanno et al. criteria [40]. Although most of the inbred lines (62 %) showed an inferred ancestry higher than 60 % to one of the k = 9 ideal populations, the other 38 % of individuals showed substantial admixture (Additional file 2).

### Association analysis

The MLM accounts for multiple levels of relatedness, including population structure (**Q** or **P** matrixes) and kinship relationships (**K** matrix). The **K** matrix was calculated following Bernardo [41], as $K_{\mathrm{ij}}^{T}=\frac{S_{\mathrm{ij}}-1}{1-T}+1$. The *T* parameter represents the probability that two alleles are alike in state, given that they are not identical by descent. In practice, *T* is unknown, and should be assessed, usually, by a maximum likelihood estimation procedure. However, despite several *T* values ranging from 0.2 to 0.8 were empirically tested to evaluate its effect on results, the latter were unaffected by changes of *T*. The *P*-values of the association analysis using either the **P** matrix (based on PCO) or the **Q** matrix (based on the Bayesian approach of STRUCTURE) and two *T* values for the kinship matrix were found to be highly stable (Table 2). A significant association was found between *HaRIC_B* and SHR incidence (*P* < 0.01, Table 2), with the maximum family wise error rate being below 14 % for the number of genes analyzed and a significance level of 0.01.

**Table 2:** ** *P* -values of associations between candidate genes and SHR incidence in sunflower**

**SUNFLOWER**	**P MATRIX**		**Q MATRIX**	
**GENE ID**	**(T = 0.2)**	**(T = 0.8)**	**(T = 0.2)**	**(T = 0.8)**
** *HaMT* **	0.8400	0.8356	0.8237	0.8192
** *HaPRP2* **	0.9493	0.9225	0.4897	0.9136
** *HaCytP450C* **	0.7856	0.7820	0.4897	0.7488
** *HaROPGEF2* **	0.9142	0.9486	0.9087	0.9454
** *HaPP2C* **	0.7153	0.7256	0.6797	0.6918
** *HaRIC_A* **	0.2094	0.2112	0.2015	0.2036
** *HaRIC_B* **	0.0098**	0.0096**	0.0066**	0.0066**
** *HaHP2* **	0.5818	0.5993	0.7514	0.5113
** *HaZFHD* **	0.4526	0.4539	0.3443	0.3472
** *HaGDPDI* **	0.9807	0.9811	0.9770	0.9775
** *HaWP* **	0.4699	0.4645	0.4501	0.4449
** *HaTRP* **	0.3764	0.3707	0.3501	0.9142
** *HaPAL* **	0.1635	0.1646	0.1342	0.1359
** *HaWRKY5* **	0.2255	0.2249	0.1398	0.1408
** *HaWRKY7* **	0.1069	0.1019	0.0933	0.0892
** *HaGLP4* **	0.3487	0.3567	0.3128	0.3219

Kinship relationships were obtained as suggested by Bernardo [41]. The K matrix was calculated as $K_{\mathrm{ij}}^{T}=\frac{S_{\mathrm{ij}}-1}{1-T}+1$ , where *T* is the probability that two alleles are alike in state, given that they are not identical by descent. In practice, *T* is unknown, but different *T* values (0.2, 0.3, 0.7, 0.8, only showing data for 0.2 and 0.8) were evaluated to reach the maximum likelihood in model (1) explained in Methods Section. **P < 0.01.

**Figure 2:**
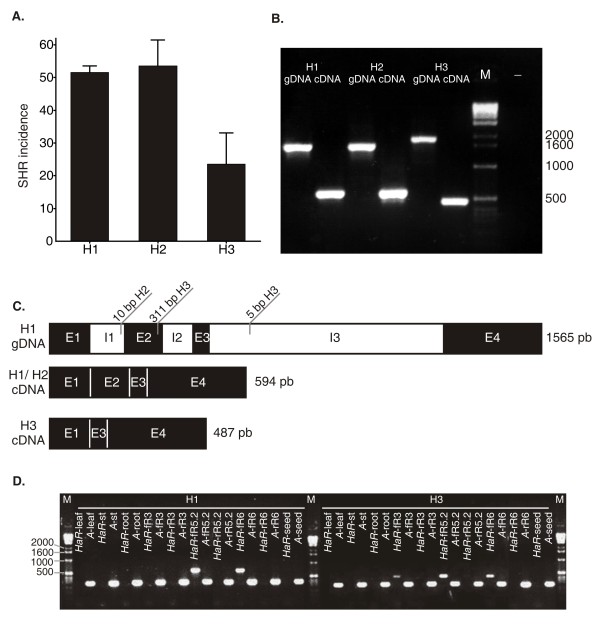
** *HaRIC_B* characterization. A**. SHR incidence means for inbred lines carrying *HaRIC_B* haplotypes 1, 2 and 3 (H1, H2, H3). Error bars refer to standard error. **B**. PCR amplification of *HaRIC_B* using genomic (gDNA) and cDNA of inbred lines carrying *HaRIC_B* haplotypes 1, 2 and 3, respectively. **C**. Gene structure, polymorphisms found in haplotypes 2 or 3 compared to haplotype 1, and splicing patterns of *HaRIC_B* for the different haplotypes found in the AMP. **D.** Expression of *HaRIC_B* haplotypes 1 (H1) and 3 (H3) in different plant organs. RT-PCR for *HaRIC_B* (*HaR*) and *Actin* (*A*) for leaf (leaf), stem (st), root (root), florets at R3 (fR3), receptacle at R3 (rR3), florets at R5.2 (fR5.2), receptacles at R5.2 (rR5.2), florets at R6 (fR6), receptacle at R6 (rR6) and dry seeds (seed).

In order to identify which haplotype was involved in the association detected, the averages of the SHR incidence adjusted means were plotted against the three different haplotypes found in *HaRIC_B* (Figure 2A). The haplotype 3 is associated with the lowest levels of SHR incidence and is present in four inbred lines (GP365, C192-1, 5381 and 5393-E), which showed SHR incidence of 0.0, 22.5, 25.5 and 46.3 %, respectively. The effect of haplotype 3 was estimated as an incidence reduction of 19 % relative to the average incidence estimated for the whole set of inbred lines involved in the experiment.

*HaRIC_B* PCR amplification using genomic DNA from inbred lines carrying haplotypes 1, 2 and 3 yielded products of different lengths. Haplotype 1 generated the shortest PCR product (1565 bp), haplotype 2 produced an intermediate length fragment correlating with a 10 bp insertion present in the first intron (1575 bp), and haplotype 3 amplified the longest PCR product (1871 bp, Figure 2B and 2 C). *HaRIC_B* was also analyzed at the cDNA level to investigate its gene structure in sunflower. According to this analysis, *HaRIC_B* genomic structure consists of four exons and three introns (Figure 2C). Haplotype 1 and 2 did not show length variation at the cDNA level. The length difference between haplotype 1/2 and 3 corresponds to a 311-bp insertion located 31 bp before the end of exon two and to a 5-bp indel detected in the third intron (Figure 2C). The 311-bp insertion leads to the splicing of exon two and to a shorter RT-PCR product (487 bp vs. 596 bp, Figure 2C). At the protein level, this splicing pattern causes a frame-shift which results in a premature stop codon (data not shown). Accumulation of *HaRIC_B* transcripts was examined by RT-PCR in different organs and capitula developmental stages (R3, R5.2, R6) of inbred lines carrying haplotypes 1 and 3, respectively. *HaRIC_B* is accumulated specifically in florets, in contrast to the housekeeping gene, which was found in all the tested tissues (Figure 2D).

## Discussion

The genetic determinants of SHR resistance were investigated following a candidate gene approach intended to provide fine-scale resolution to QTL mapping efforts for this trait. Selection of candidate genes is one of the fundamental challenges of candidate gene AM, particularly when there is limited knowledge about the molecular mechanisms underlying the trait under study. For SHR, the most comprehensive experimental evidence comes from the study of gene expression changes in two genotypes of the oilseed *B. napus* infected with *S. sclerotiourum* using a whole genome microarray from *A. thaliana*[25]. However, transferring this information to sunflower was hindered by ortholog identification between highly divergent species. Commonly, most researchers use pairwise distance comparisons algorithms, such as BLAST, COG (Clusters of Orthologous Groups), RBH (Reciprocal BLAST Hits), RSD (Reciprocal Smallest Distance Algorithm) and INPARANOID, to determine gene orthology [42]. In contrast, although they have been shown to exhibit greater accuracy and lower error rates than pairwise comparison methods, phylogeny based approaches have been only partially exploited due to the complexity of the automation of sequence alignment and the choice of appropriate genes and species to be included in the analysis [42,43]. Notwithstanding, it is only by phylogenetic reconstruction that ortholog and parolog relationships can be distinguished, especially for species with incomplete genome sequence data where the best hit of pairwise comparison methods is often not the nearest neighbor [44].

The phylogenetic approach devised here allowed identification of 17 genes from Asteraceae that are orthologs or paralogs to the *A. thaliana* loci detected as over-expressed at 24 hpi in the *B. napus* resistant genotype. Particularly, for At5g51550, At3g48310, At5g05940, At1g04450 and At1g13200, two to three loci were identified as paralogs to the *A. thaliana* sequence used as query, providing wider coverage of their putative functional spectrum (Table 1).

Two additional sources served to increase to 30 the number of candidate genes, an EST library obtained through suppressive subtractive hybridization from sunflower capitula of the genotype RHA801 infected with *S. sclerotiorum* at 48 hpi, and previous literature reports [36-38]. After identification of polymorphisms (SNPs and indels) within the core set of 10 inbred lines, a total of 21 candidate genes were selected to be further genotyped in the AMP. This number of candidate genes has proved adequate to find significant genotype-phenotype associations for traits with different degrees of complexity. Examples include the studies carried out in *A. thaliana* for flowering time [45], in potato for late blight [30] and in maize for aluminum tolerance [31]. Moreover, in this study the authors used information sources similar to those described here to select the candidate genes, finding significant associations for six of them.

The AMP analyzed here is representative of the elite breeding pool used by INTA in the “Sunflower Breeding Program”. It encompasses germplasm from different geographical origins, with some of the lines being derived from introgressions with wild Helianthus species (Additional file 1). In agreement with the morphological diversity and intricate ancestry of the AMP, the SSR markers revealed high levels of genetic variability. Indeed, the mean number of alleles per locus was even higher than the estimate obtained for a set of compounds, populations and lines conserved at the INTA Germplasm Bank (6.6 vs. 5.71) [46].

As suggested by Stich et al. [47], marker-trait associations were assessed using MLM, which took into account trials, blocks, population structure and kinship relationships to control type I error rates. Both PCO and the Bayesian method (STRUCTURE software) were used to infer population structure for the 94 elite inbred lines. While PCO analysis showed no clear grouping pattern by drawing the two first Principal Coordinates, STRUCTURE suggested the presence of nine different gene pools (Additional file 2). The lack of defined groups in PCO is not unexpected given the restricted genetic of cultivated sunflower [1]. However, either using **P** or **Q** matrixes in the association analysis did not have an impact on the *P*-values (Table 2). Moreover, varying the probability *T* for the calculation of the **K** matrix did not modify the association results.

A significant association was detected between a lower SRH incidence and the haplotype 3 of *HaRIC_B* (*P* < 0.01). The nature of the mutation found in the haplotype 3 of *HaRIC_B*, the highly specific expression pattern for this gene in sunflower and the experimental evidence on the role of this protein family in *A. thaliana*[48,49] lend support to the biological significance of the detected association. *HaRIC_B* was selected from the study of Zhao et al. [25] following ortholog identification via phylogenetic analysis. Haplotype 3 showed an insertion of 311-bp at the 3’ end of exon two, which alters the RNA splicing and, consequently, leads to both the generation of a frame-shift and a premature stop codon in the mRNA. Only *ca.* 15 % of mutations that have been identified as being associated with genetic variation in plant quantitative traits involve changes in the amino acid composition of proteins. However, it has been shown that many of the associations that correspond to noncoding mutations located in introns, untranslated regions or intergenic regions show up as significant because they are in LD with untyped causal mutations that in turn are nonsynonymous substitutions [50].

Although *HaRIC_B* molecular function has been inferred by homology, the orthologous *A. thaliana* gene (AT1G04450, RIC3) has been experimentally described as a small binding-protein that interacts with ROP1 (Rho family GTPase). There are 11 Arabidopsis RIC proteins (for Rop-interactive CRIB motif–containing proteins) involved in pollen tube growth and other functions [49]. Wu et al. [49] have shown that RIC3 transcripts are found only in the flowers and inflorescences of *A. thaliana*. In agreement with its proposed ortholog relationship, the expression pattern of *HaRIC_B* matches that found for RIC3, with transcripts being present in florets from R3 to R6 developmental stage (Figure 2D). It is noteworthy that R5.2 is the period of maximum susceptibility to *S. sclerotiorum* infection in the sunflower cultivation areas of South America, and florets are the main entry point for the pathogen [4].

Different studies suggest that the role of RIC3 involves elevation of cytosolic Ca^2+^ and regulation of (1) de-polymerization of actin filaments, (2) exo-cytosis, and (3) Ca^2+^ mediated signals [48,51]. Interestingly, the Rop-interactive domain of *HaRIC_B* is located in exon two, which is missing in plants carrying haplotype 3. Thus, if a functional protein can be synthesized from it, its regulation by Rop GTPases would seemingly be not possible.

Recently, two necrosis and ethylene-induced peptides (*NEPs*) have been described in *S. sclerotiorum* (*SsNep1* and *SsNep2*). *SsNep2* expression is highly dependent on Ca^2+^ concentration, and compounds increasing calcium levels (i.e. caffeine and lanthanum chloride) greatly reduced *S. sclerotiorum* virulence and expression of *SsNep2*[52]. Thus, consistent with its putative role of intracellular calcium elevation, the haplotype 3 of *HaRIC_B* might be participating in the defense against SHR by repressing expression of the necrosis factor *SsNep2*, through the de-regulation and elevation of cytosolic Ca^2+^ concentrations in the target organ for pathogen attack.

The power of association mapping greatly depends not only on the allele frequency distribution but also on the magnitude of the effect that can be ascribed to a locus, relative to other loci present in the population [53]. Thus, the detection of association between *HaRIC_B* (haplotype 3) and a lower SHR incidence, despite the fact that haplotype 3 was in low frequency, suggests that it has a strong effect on the phenotype. Indeed, considering that resistance is the result of the interaction of multiple factors, having found a single haplotype that accounts for a SHR incidence reduction of about 20 % in average, emphasizes the importance of the finding. However, beyond the experimental evidence presented here, and the biological considerations that support the role of haplotype 3 on the resistance to SHR, validation of these results will require the re-evaluation of this candidate polymorphism in a larger AMP and different field testing environments. A wider association study is currently underway using large-scale gene sampling and high-throughput genotyping methods.

It has been shown that the causal polymorphism for a QTL can be distant from the functional gene under analysis, particularly in species with high levels of LD, such as sunflower [33-35,54]. While it cannot be ruled out that the polymorphism responsible for a lower SHR incidence resides in a linked ungenotyped region, the evidences discussed here suggest that *HaRIC_B* can be considered a strong candidate to be directly involved in SHR resistance. To assess its relationships with QTL previously identified in biparental populations, *HaRIC_B* was genotyped in the sunflower RIL mapping population PAC2 X RHA266. It was mapped to LG11 between the markers E36M59_9 and E38M50_17 (data not shown), a region for which no QTL have been reported to date [3,7,9-14,55]. This is not unexpected, as the parental lines used to investigate SHR resistance may not carry the haplotype 3 of *HaRIC_B*, especially considering its low frequency in the AMP. In this context, *HaRIC_B* may be thought of as a new and highly delimited QTL for SHR resistance.

## Conclusions

Identification of genes and alleles underlying resistance to SHR has been a major concern for sunflower research groups. The present work contributes to previous QTL studies by identifying the most highly delimited QTL for SHR reported to date. Validation experiments are currently ongoing to determine the specific role of *HaRIC_B*, however, the association found is highly supported by (1) its specific expression pattern in the target organs of *S. sclerotiorum* infection, (2) the role of its orthologous gene in Arabidopsis in altering cytosolic Ca^2+^ concentration, (3) the fact that Ca^2+^ plays an important part in reducing *S. sclerotiorum* virulence. Finally, our results demonstrate that association genetics via candidate genes is a valuable approach for elucidating the molecular basis of complex agronomic traits in sunflower, and for developing DNA-based markers for “precision breeding” of improved varieties.

## Methods

### Plant material

The sunflower Association Mapping Population (AMP) was composed of 94 inbred lines. Ten of them are public lines and have been previously used in the development of biparental populations, while the remaining 84 belong to the “Sunflower Breeding Program” of INTA. The inbred lines are described in Additional file 1 and additional information is available from the Sunflower Germplasm Bank.

### Genomic DNA extraction

The DNA was extracted with NucleoSpin® Plant II or NucleoSpin® 96 Plant II (Macherey-Nagel, Argentina) from lyophilized leaves from 3-week old plants grown in the experimental field.

### Field trials

Field experiments were conducted at Balcarce Experimental Station-INTA (37° 50′ 0″ S, 58° 15′ 33″ W, Province of Buenos Aires, Argentina) during growing seasons 2008/2009 (sowing date November 21^st^, 2008) and 2009/2010 (sowing date December 11^th^, 2009). Seeds were sown in typical Argiudol soil containing 5 % of organic matter at pH 6.2.

A randomized complete block design with two replications was used. Each experimental unit had one row 6.0 m length by 0.7 m wide, containing 30 plants. Sunflower capitula were sprayed with inoculum (1 cm^3^) at the R5.2 stage from the Schneiter and Miller’s scale [56]. Capitula were immediately covered with paper bags up to 15 days post inoculation. A control to check the efficacy of the inoculation procedure (one susceptible cultivar inoculated at once with the tested inbred lines) was included. SHR incidence, i.e. the percentage of infected capitula in each row was evaluated at R9 stage [56].

### Inoculum preparation

A population of *S. sclerotiorum* from Balcarce (Buenos Aires, Argentina) was used for inoculum preparation. The sclerotia were collected in the field and stored in paper bags at 13° ± 5° for three months. For ascospore production, Escande et al. [57] procedure was followed. Shortly, sclerotia were exposed at -18°± 2° for seven days and buried 1 cm deep in humid pasteurized soil until stile emergence. Cultures were incubated at 16° and approximately 2500 lux of continuous daylight. Mature apothecia were harvested and positioned upside down in glass Petri dishes for 4 h to favor ascospore releasing. Ascospores were stored in Petri dishes at -18° until use. For capitula inoculation, ascospores were suspended in water to a concentration of 2500 spores/cm^3^.

### Candidate gene selection

Candidate genes for SHR resistance were selected from three different data sources: (1) 19 loci, at least twofold over-expressed in a *B. napus* resistant genotype after 24 hours post-infection (hpi), were selected from the study of Zhao et al. [25]; (2) 17 candidate genes were selected according to their annotated function (e.g. biotic and abiotic stress related proteins, cell wall and cell membrane proteins) from an EST library obtained through suppressive subtractive hybridization from capitula of the genotype RHA801 infected with *S. sclerotiorum* at 48 hpi [38]; (3) seven candidate genes, i.e. two WRKY transcription factors and five germin-like proteins, previously identified in sunflower and reported to participate in the defense response against *S. sclerotiorum* infections in sunflower and other species were also selected [36,37]. The general features of the candidate genes are described in Additional file 3.

In the analysis of Zhao et al. [25], the transcriptional differences were detected using a 70-mer oligo-microarray from *Arabidopsis thaliana*, therefore, a phylogenetic approach was devised to identify the corresponding orthologous genes in sunflower. Briefly, a tBLASTn search [58] was performed against several data bases (GenBank Viridiplantae EST data base, KEGG *Helianthus annuus* data base, DFCI Sunflower Gene Index) using as query the *A. thaliana* protein sequences. A group of EST sequences with E-values < 10^-20^ and high similarity to each query (≥ 50 % of identity in amino acid sequence) was selected to represent different plant families, and was used to determine the position and minimum length of the regions to be included in the phylogenetic reconstruction. ESTs with hits over two regions with different Open Reading Frames (ORF) were excluded from the analysis. All alignments were required to include at least 150 amino acids. To avoid over representation of particular groups, a maximum of four ESTs was allowed per species, except for species of the genera Helianthus and Lactuca, for which all detected hits were considered for analysis. Once the sequences were selected, they were translated into the appropriate ORF and aligned using the MAFFT routine L-INS-i [59]. The protein alignments were used to obtain the phylogenetic relationships through Maximum Parsimony (MP) using the software TNT [60]. Heuristic searches were performed starting from 20 Wagner trees with tree bisection-reconnection branch-swapping (TBR). All characters were treated as equally weighted. Only when a low number of replicates found the shortest trees, an additional TBR branch-swapping search over the trees found previously was performed. Node support values in MP analyses were assessed using 100 jacknife replicates. Finally, sunflower orthologous and paralogous sequences were determined based on the phylogenetic relationships found for each locus. Orthology was assigned comparing gene trees with species trees to infer speciation/duplication events. In the absence of sunflower sequences orthologous to the *A. thaliana* query, sunflower paralogous and/or orthologous sequences from either Lactuca or other Asteraceae species were selected for subsequent analysis. The sequences selected for each locus were used as template for primer design.

### Primer design and PCR amplification

For those candidate genes for which *H. annuus* sequences were available, primer design was conducted using software Primer3 [61]. In those instances in which only Lactuca or other Asteraceae sequences were available, degenerated primers were designed either manually or using the software iCODEHOP [62].

Specific amplification products obtained with degenerated primers were purified with QIAquick PCR Purification Kit (QIAGEN, Germany). Afterwards, they were cloned on pGEMT-easy (Promega, USA) and at least ten colonies were sequenced. Sequences that yielded high similarity by BLAST searches [58] to the target sequence were used to design new non-degenerated primers with software Primer3 [61].

Primer functionality was assayed using RHA801 and HA89 genomic DNA as templates in the PCR amplification. PCR reactions were performed as described previously in Fusari et al. [35] using touchdown programs ranging from 68° to 55°.

Primer pairs yielding the PCR products of expected sizes were then used to amplify a total of ten inbred lines, the CS, to detect polymorphisms (SNPs and indels). PCR products were purified either as described previously or using ExoSAP (Exonuclease I & Shrimp Alkaline Phosphatase, USB, USA). Sequencing reactions, sequence analysis and polymorphism identification were done as described in Fusari et al. [35].

### Genotyping of candidate genes in the AMP

DNA polymorphisms found in the CS were used to define haplotypes for each candidate gene (Table 1). According to the complexity and number of haplotypes in each case, different genotyping methods were selected. When needed, new primers were designed with Primer3 software [61] for genotyping purposes. Those genes which showed only two haplotypes in the CS were genotyped with denaturing dHPLC, those which had indels were genotyped by FCE and those with more than two haplotypes determined by both SNPs and indels were genotyped by direct sequencing (Table 1). The dHPLC and FCE genotyping methods were carried out as described in Fusari et al. [63]. For dHPLC, PCR products that did not match any of the haplotypes tested or displayed a heteroduplex-like profile in the homoduplex controls were subjected to direct sequencing. For FCE, PCR products of different length than those found in the CS were amplified with unlabeled primers and sequenced. All sequencing reactions and SNP analyses were performed as described previously [35].

In all cases, three individuals per inbred line were genotyped separately, making a total of 282 genotyped individuals.

### Genotyping of SSR in the AMP

To study the genetic structure of the AMP, eight SSR loci located in different linkage groups of the sunflower genetic map were chosen from a preliminary survey of 35, based on their power to distinguish among individuals [46]. The selected loci (HA77, HA293, HA928, HA991, HA2063, HA2920, HA3239, HA4103) were genotyped in the 94 lines of the AMP (3 individuals per inbred line, 282 individuals in total) using FCE as previously described [63].

LG position and size data of SSR loci are available in Poormohammad Kiani et al. [64]. Although most of the lines were highly inbred, less than 10 % of the accessions exhibited a small proportion of heterozygosity. In such cases, a consensus genotype was established for the corresponding loci-line combination based on the genotypes of the three individuals scored. The criteria applied to designate the consensus genotype was as follows: (a) if two individuals carried the same homozygous genotype and the third individual was heterozygous carrying the allele present in the other two individuals, the inbred line was considered homozygous for such allele; (b) if two individuals were heterozygous and the third one was homozygous for any of the alleles detected in the other individuals, the inbred line was scored as heterozygous for those alleles; (c) if two out of three individuals were homozygous for different alleles, the inbred lines was considered heterozygous for those alleles. In those loci for which more than three alleles were present among the individuals, no consensus genotype was determined, and it was considered as missing data. The probability of identity (PI) and the probability of identity considering the genetic similarity among sibblings (PI sibs) were computed from the AMP data as described by Waits et al. [65] using GenAlEx6 [66]. These statistics are widely used as an indication of the minimum number of loci required for reliable genetic tagging [66]. The estimates obtained for the present AMP (PI = 0.000006; PI sibs = 0.004) are well within the ranged accepted in population genetics studies [65].

### Statistical analysis

The statistical analysis was conducted according to the two-step method described in Stich et al. [47]. Haplotype-based tests were preferred to single-marker-based tests to increase power to detect associations.

The phenotypic data were analyzed on the basis of a Mixed Linear Model (MLM) encompassing inbred lines as fixed effect and the “trial” and “blocks within trials” as random effects:

(1) $${\text{Y}}_{\mathrm{ijk}}=\mu +\text{Lin}e_{i}+\text{Tria}l_{j}+\text{Blocks}{\left(\text{Trial}\right)}_{\mathrm{jk}}+{\text{e}}_{\mathrm{ijk}}$$

where $Y_{\mathrm{ijk}}$ represents Sclerotinia Head Rot incidence at the R9 stage, *μ* a common mean value for every observation, *Line*_*i*_ the effect of the i*th* inbred line, *Trial*_*j*_ the random effect of the j*th* trial (2008/2009 and 2009/2010), *Block (Trial)*_*jk*_ the random effect of the k*th* block in the j*th* trial and e_*ijk*_ the random error of each observation.

Over the two trials, the adjusted inbred line mean was calculated for the 94 inbred lines:

(2) $${\hat{M}}_{i}=\hat{\mu }+\hat{L}ine_{i}$$

The association analysis was performed with a MLM, where the adjusted inbred line mean was modeled for the j*th* candidate gene by:

(3) $$M=\mu 1+Xm^{\left(j\right)}+\left(P\;or\;Q\right)\beta +Ub+e$$

Where **M** is the vector of length *ℓ* (# inbred lines) of estimated SHR incidence (94 adjusted means), μ is the common mean, **1** is a *ℓ*-length vector of ones, **X** is an *ℓ* x h_j_ incidence matrix, **m**^*(j)*^ is the haplotype effect vector of length h_j_, of the j^*th*^ candidate gene, **P** (or **Q**) are *ℓ* x *p* matrixes which account for the population structure, **β** is a *p*-length vector of regression coefficients associated to **P** (*or***Q**) and **U** is a *ℓ* x *ℓ* matrix of weights for the *ℓ*-length vector random effects **b** of inbred lines effects such as **K** = **UU’**. The assumptions for the random components of the model are: **b** ~ *N* (**0**, σ_e_^2^** *K* **) being **K** the kinship matrix between inbred lines, **e** ~ N (**0**,σ_e_^2^** *I* **), and cov(**b**,**e**) = **0**.

Population structure was taken into account by two different approaches: Principal Coordinates Analysis (PCO), which generates the **P** matrix (94 inbred lines x 5 Principal Coordinates), and the Bayesian method implemented in STRUCTURE software, which generates the **Q** matrix (94 inbred lines x membership coefficients to each of the 9 ideal populations detected) [39].

PCO was performed based on Roger’s distance [67] obtained from the SSR allele frequency matrix (94 inbred lines x 47 alleles) using InfoStat software [68]_._ The first five principal coordinates, which explain altogether 43 % of the variance, were used in the **P** matrix.

Population structure matrix **Q** was calculated with STRUCTURE software [39]. The number of k populations evaluated ranged from 0 to 12. The analysis was performed using five replicates per k, a burn-in period of 10^5^ and a run length of 5*10^5^. Allelic frequencies were kept correlated. No prior information on the origin of individuals was used to define groups. The run showing the highest posterior probability was considered for each k value. Estimation of the number of populations (k) was conducted following the software documentation and the *ad hoc* criterion proposed by Evanno et al.[40].

Kinship relationships among inbred lines were accounted for by the **K** matrix (94 x 94) proposed by Bernardo [41]. The matrix K is defined as $K=\left\{K_{{}_{\mathrm{ij}}}^{T}\right\}$, where $K_{\mathrm{ij}}^{T}=\frac{S_{\mathrm{ij}}-1}{1-T}+1$, S_*ij*_ is the proportion of shared alleles (SSR) between inbred lines *i* and *j,* and *T* the probability that two alleles are alike in state, given that they are not identical by descent. In practice, *T* is unknown, but different *T* values (0.2, 0.3, 0.7, 0.8) were evaluated to assess their effect on the likelihood of model (1). All MLM estimations were performed with the *lme* routine of R software [69].

FDR procedures to adjust *P*-values were not applied for three reasons: (a) we were working with a small number of genes which were selected *a priori* as candidates to be associated with the disease, (b) the inclusion of **P** (or **Q**) and **K** matrixes in the model has the purpose to control for the occurrence of false positives due to population structure and kinship relationships, and (c) FDR procedures tend to produce high false-negative error rates. Notwithstanding the maximum family wise error rate was estimated as a reference.

### Characterization of genes associated to SHR resistance

Seeds from three inbred lines showing *HaRIC_B* haplotypes 1, 2 and 3, respectively, were grown in the greenhouse. Leaves, stems and roots were harvested in liquid nitrogen at V8 developmental stage. Additionally, florets and receptacles of sunflowers at R3, R5.2 and R6 stages were harvested in liquid nitrogen separately. The RNA was extracted with RNAqueous® kit (Ambion®, USA), according to manufacturer’s instructions. To remove polysaccharides and polyphenolics the tissue was homogenized in a mixture of RNA Isolation Aid (Ambion®, USA) and RNAqueous® Lysis/Binding solution. One μg of DNAse-treated RNA was used to perform RT-PCR with the SuperScript III Reverse Transcriptase (Invitrogen, USA) using random primers according to the kit instructions.

To determine *HaRIC_B* gene structure, genomic DNA and cDNA from R5.2 capitula of inbred lines carrying haplotypes 1, 2 or 3 were used as template in PCR. To determine *HaRIC_B* expression pattern cDNA from leaf, stem, root, dry seeds, florets and receptacles from capitula at the R3, R5.2 and R6 developmental stages were used for the RT-PCR. Amplifications were carried out as previously described using a touchdown program (60°–55°) with primers F: 5′ TTGAGGGATTCTAATTGTTATAGTTGA 3′ and R: 5′ TTCGGGTGTTCGTCCTTTT 3′ for *HaRIC_B* and F: 5′ GGAGCAGAGAGATTCCGTTG 3′ and R: 5′ GAAGGTGCTGAGTGATGCAA 3′ for *Actin*.

## Competing interests

The Authors declare no competing financial interests.

## Authors’ contributions

CMF selected the candidate genes along with VVL and NBP. CMF amplified the regions, carried out SNPs and indel development in the CS and performed candidate gene genotyping of the AMP. CT, CM and FQ planned and coordinate the phenotypic trials. CT collected the phenotypic data. DA selected the inbred lines to be included in the AMP. MVM and VN performed microsatellite genotyping. CMF and VL performed STRUCTURE and PCO analyses. JDR implemented the MLM and supervised the statistical analysis. RAH provided EST sequence information. CMF, VVL and NBP accomplished data and results interpretation. CMF, VVL and NBP wrote the manuscript. RAH und JDR helped to draft the manuscript. VVL and NBP coordinated the study. HEH and AE initiated the project and contributed to the work by the interpretation and discussion of the data. All authors read and approved the manuscript.

## Authors’ information

Dr. VVL; Dr. RAH and Dr. NBP are career members of the Consejo Nacional de Investigaciones Científicas y Técnicas (CONICET). Dr. HEH is a career member of the Comisión de Investigaciones Científicas de la Provincia de Buenos Aires (CIC). CMF is current Alexander Von Humboldt Foundation’s fellow at the Max Planck Institute of Molecular Plant Physiology (Germany).

Verónica V. Lia and Norma B. Paniego co-directed the work and share last authorship.

Sequence Data is available at GenBank: **JN231331** to **JN231540**.

## Supplementary Material

Additional file 1 Inbred Lines used for Association Mapping in Sunflower and Adjusted Means for Sclerotinia Head Rot Incidence. Click here for file

Additional file 2 **Analyses of population Structure in the AMP. A.** Principal coordinate analysis of the AMP based on Rogers’ distance estimates. Percentages in parentheses refer to the proportion of variance explained by the principal coordinate (PC). **B.** Data posterior probability (Ln x/k) and rate of change in the log probability of data between successive k values (Δk). The values for k = 9 are indicated by arrows. **C.** Estimated population structure at k = 9. Each inbred line (bar) is partitioned into k colored segments that represent the individual’s estimated membership fractions in k clusters. Click here for file

Additional file 3 Candidate Genes Selected for Sclerotinia Head Rot Resistance. Click here for file
