# Supplementary figures and images for: Association mapping in sunflower for sclerotinia head rot resistance
Source: BMC Plant Biol. 2012 Jun 18;12:93. doi: 10.1186/1471-2229-12-93 (PMC3778846; doi:10.1186/1471-2229-12-93)

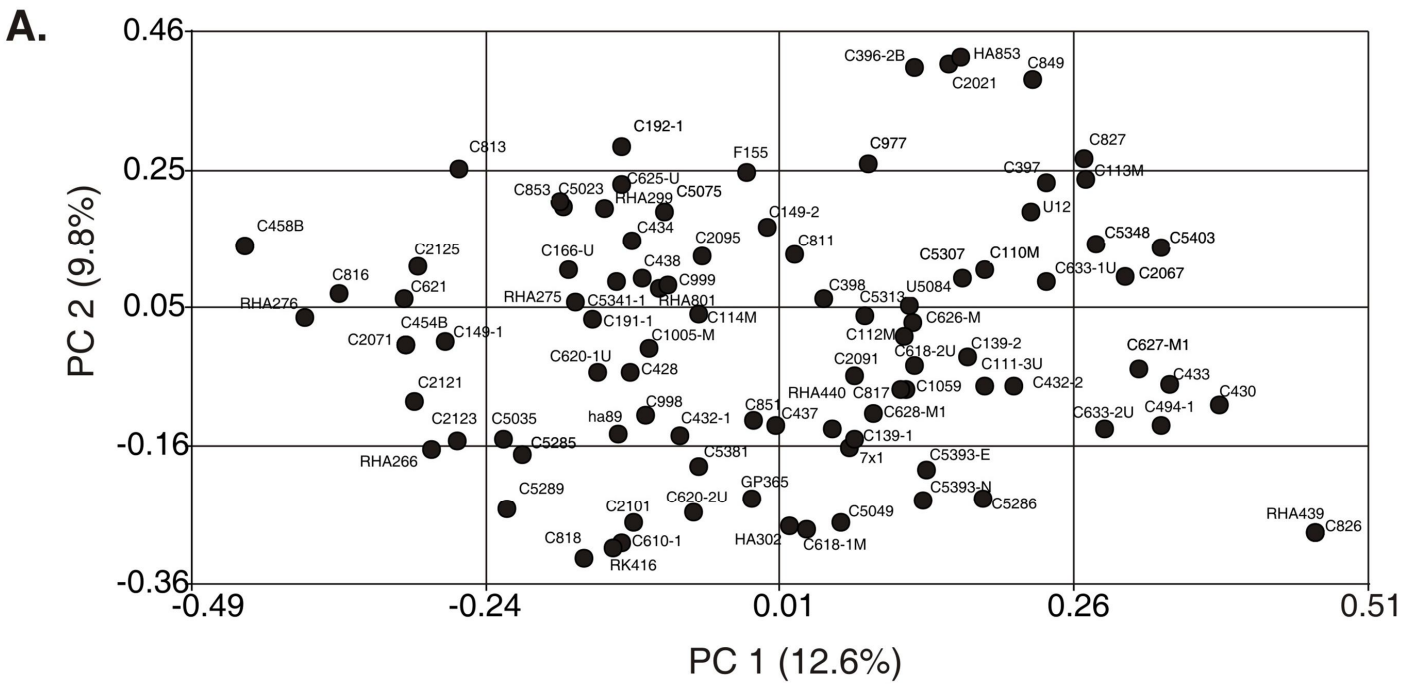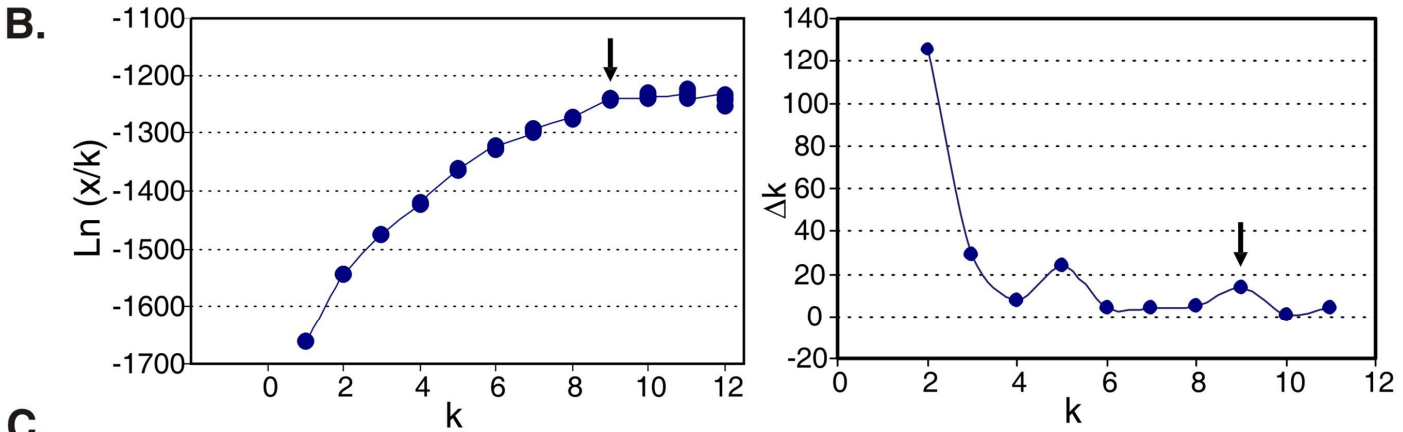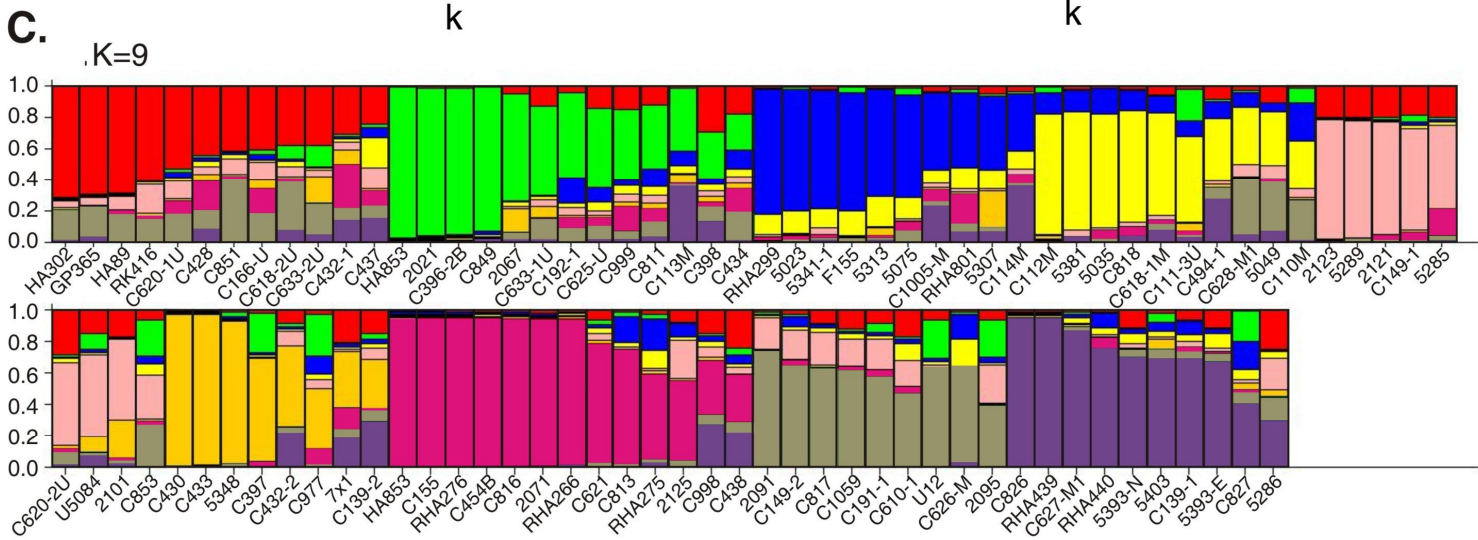

Supplement: Additional file 2 — Analyses of population Structure in the AMP. A. Principal coordinate analysis of the AMP based on Rogers’ distance estimates. Percentages in parentheses refer to the proportion of variance explained by the principal coordinate (PC). B. Data posterior probability (Ln x/k) and rate of change in the log probability of data between successive k values (Δk). The values for k = 9 are indicated by arrows. C. Estimated population structure at k = 9. Each inbred line (bar) is partitioned into k colored segments that represent the individual’s estimated membership fractions in k clusters. [file 1471-2229-12-93-S2.pdf]
